# A Deep Transfer Learning Framework for Speed-of-Sound Aberration Correction in Full-Ring Photoacoustic Tomography

**DOI:** 10.3390/s26020626

**Published:** 2026-01-16

**Authors:** Jie Yin, Yingjie Feng, Qi Feng, Junjun He, Chao Tao

**Affiliations:** 1 School of Electrical and Control Engineering, Nanjing Polytechnic Institute, Nanjing 210048, China; 2 MOE Key Laboratory of Modern Acoustics, Department of Physics, Collaborative Innovation Center of Advanced Microstructures, Nanjing University, Nanjing 210093, China; fyj010825@163.com; 3 School of Electronic Information and Electrical Engineering, Shanghai Jiao Tong University, Shanghai 200240, China; qfeng.med@gmail.com; 4 School of Intelligent Manufacturing, Nanjing Polytechnic Institute, Nanjing 210048, China; hejunjun@njpi.edu.cn

**Keywords:** photoacoustic tomography, image reconstruction, deep transfer learning, speed-of-sound, aberration correction

## Abstract

Speed-of-sound (SoS) heterogeneities introduce pronounced artifacts in full-ring photoacoustic tomography (PAT), degrading imaging accuracy and constraining its practical use. We introduce a transfer learning-based deep neural framework that couples an ImageNet-pretrained ResNet-50 encoder with a tailored deconvolutional decoder to perform end-to-end artifact correction on photoacoustic tomography reconstructions. We propose a two-phase curriculum learning protocol, initial pretraining on simulations with uniform SoS mismatches, followed by fine-tuning on spatially heterogeneous SoS fields, to improve generalization to complex aberrations. Evaluated on numerical models, physical phantom experiments and in vivo experiments, the framework provides substantial gains over conventional back-projection and U-Net baselines in mean squared error, structural similarity index measure, and Pearson correlation coefficient, while achieving an average inference time of 17 ms per frame. These results indicate that the proposed approach can reduce the sensitivity of full-ring PAT to SoS inhomogeneity and improve full-view reconstruction quality.

## 1. Introduction

Photoacoustic tomography (PAT) leverages optical absorption contrast in biological tissue and ultrasonic (US) detection to produce high-resolution images. Over the past decade, PAT has matured into a promising clinical diagnostic modality [1,2,3,4,5]. In PAT, short laser pulses illuminate the target and generate photoacoustic (PA) waves, which are recorded by ultrasound transducers surrounding the tissue. Reconstruction algorithms then recover the initial pressure distribution, which is directly related to local optical absorption [6,7]. Recent advances in PAT have demonstrated kilohertz-scale volumetric imaging rates that far surpass those of conventional modalities, and have enabled visualization of rapid physiological processes that were previously inaccessible in vivo [8,9,10]. Among various geometries, full-ring PAT provides nearly complete angular coverage and supports comprehensive visualization of internal structures, making it attractive for both preclinical and emerging clinical applications [11,12,13].

The imaging quality of PAT is strongly influenced by the reconstruction algorithm. The conventional analytic reconstructions, such as back-projection (BP), usually assume a spatially invariant speed-of-sound (SoS); yet biological tissues possess heterogeneous acoustic properties, and even slight SoS inhomogeneities introduce wavefront aberrations that appear as blurring, geometric distortion, and artifacts in PAT images, severely lowering the imaging quality [14,15]. Accordingly, several approaches have been developed to mitigate SoS-induced artifacts in PAT [16,17,18]. Iterative or time-reversal methods (e.g., finite-difference time-domain or k-Wave simulations) can model heterogeneous media by solving the full wave equation [16,17]. These approaches require prohibitive computational costs that preclude real-time deployment in clinical or intraoperative contexts. Joint reconstruction techniques can simultaneously recover initial pressure and SoS maps [19,20,21,22,23,24]. However, accurate joint reconstruction may not be achievable due to the numerical instability; therefore, prior information on SoS distributions and detection geometries was usually incorporated [20]. An autofocus algorithm is proposed to determine an optimal single SoS value for the medium [25], which performs a global optimization of the SoS and neglects local heterogeneities within the tissue. These approaches either depend on prior knowledge, which is often unavailable in practical settings, or incur prohibitive computational costs, making timely deployment infeasible. Therefore, most implementations still rely on a constant SoS for image reconstruction, which seriously limits the application of PAT in complex tissues.

In recent years, deep learning (DL) has transformed medical imaging by learning complex image-formation mappings directly from data, yielding rapid, high-fidelity reconstructions in various biomedical imaging modalities such as MRI, CT, and US. Photoacoustic imaging has likewise adopted data-driven techniques, including reconstruction from sparse-view measurements [26,27], CNN-based bandwidth enhancement and sinogram super-resolution for limited-data acquisition [28], multiscale super-resolution in optical-resolution PAT [29], and human volumetric photoacoustic imaging in vivo [30]. Unlike traditional model-driven or iterative methods, DL infers the nonlinear relationship between input measurements and output images without explicit knowledge of the acoustic medium. Consequently, data-driven approaches are particularly well suited for PAT scenarios in which explicit SoS maps are unavailable or impractical to obtain, enabling artifact suppression and faithful recovery of the initial-pressure distribution. Moreover, the trained DL model requires only minimal inference time, facilitating real-time image reconstruction.

Several DL strategies have been proposed to mitigate SoS-induced aberrations in photoacoustic imaging. For example, one framework addressed scan-radius calibration errors using a classification CNN coupled with a Dense U-Net for image correction [31]. In a dual-modal PA/US system based on a clinical ultrasound probe, DL has also been used to estimate SoS and subsequently compensate aberrations [32]. Other studies have investigated joint learning of the initial pressure and the SoS distribution [33], as well as automated two-compartment SoS correction in combined US/PA CT configurations to improve isotropic reconstructions [34]. An end-to-end model has been developed to suppress aberrations from inhomogeneous SoS in linear-array PAT, yielding improved in vivo image quality [35].

Despite these advances, existing DL-based methods for correcting SoS-induced distortions still have certain limitations. The method proposed in Ref. [31] is only applicable to two specific source geometries. Ref. [33] is applicable to model-based reconstruction, which is computationally time-consuming and therefore difficult to extend to real-time imaging scenarios. Ref. [34] is designed for a two-speed (two-region) SoS distribution; however, in practical imaging scenarios, the SoS distribution often involves more than two distinct values. The approaches in Refs. [32,35] are primarily designed for linear-array PAT. However, full-ring PAT remains the definitive approach for distortion-free, full-view imaging of biological structures, but the intrinsic imaging physics of PAT render full-ring configurations inherently more sensitive to SoS heterogeneities than linear arrays, leading to amplified aberrations [36,37,38]. Ref. [39] proposed DeepMB, enabling real-time reconstruction with an adjustable global SoS. However, it does not explicitly correct aberrations induced by spatially heterogeneous SoS distributions. As a result, DL-based heterogeneous SoS distributions correction is still a challenge for full-ring arrays [40]. The goal of this study is to develop an image distortion correction method for full-ring array PAT under heterogeneous SoS distributions, while remaining computationally efficient to support real-time imaging.

Rather than training solely on specific medical imaging datasets, deep backbones pretrained on large-scale natural image corpora (e.g., ImageNet) can provide improved robustness and generalization [39]. With appropriately tailored learning strategies, such backbones can be adapted to more challenging reconstruction problems, including the mitigation of SoS-induced distortions in full-ring PAT. ResNet has shown strong performance in a wide range of medical imaging tasks [41,42,43]. Building on this, we propose a transfer learning-based framework that integrates an ImageNet-pretrained ResNet-50 encoder with a five-layer deconvolutional decoder to perform end-to-end artifact correction on full-ring PAT reconstructions. Using a pretrained ResNet-50 backbone may offer several advantages: (i) transferable low-level features (e.g., edges and local contrast patterns) that improve sample efficiency when PAT-specific data are limited, and (ii) residual connections that stabilize optimization and help preserve high-frequency structures (e.g., sharp boundaries) while learning the correction terms. To further enhance generalization, we adopt a curriculum learning scheme with two stages. In Stage I, the network is pretrained on relatively simple cases with uniform SoS offsets to learn prototypical geometric distortion patterns. In Stage II, it is fine-tuned on randomly heterogeneous SoS scenarios to specifically learn corrections under spatially varying aberrations. Numerical simulations, phantom experiments, and in vivo experiments validate the effectiveness of the proposed method in restoring image fidelity. Overall, this work introduces curriculum-based learning for aberration correction in full-ring PAT and helps mitigate the challenges posed by SoS heterogeneity in full-view tomographic imaging.

## 2. Materials and Methods

**Figure 1:**
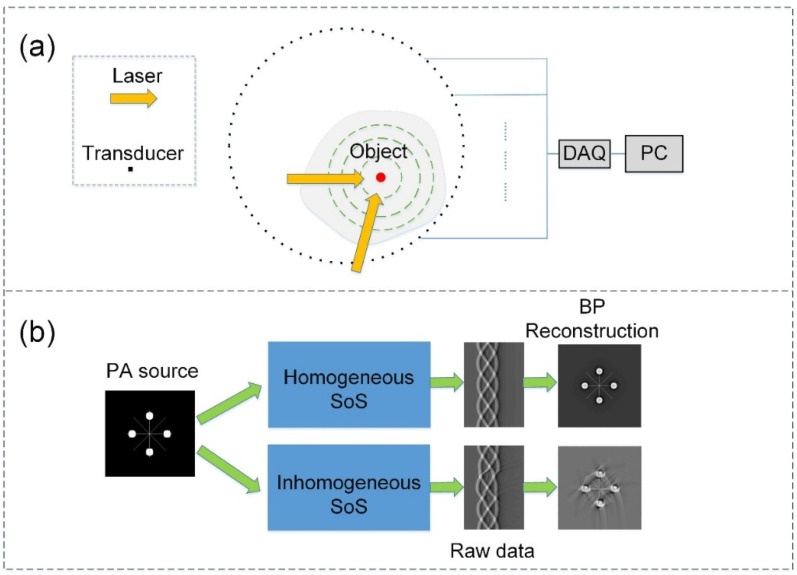
Schematic diagram of full-ring PAT in heterogeneous SoS scenario. (**a**) Schematic of the photoacoustic imaging system. The red dot denotes the PA source, the green dashed circle represents the outward propagation of the generated PA wave, and the cyan dashed lines indicate multiple omitted propagation paths of the RF signals. (**b**) Schematic illustrating the impact of heterogeneous SoS distributions on image reconstruction quality.

The basic schematic of full-ring PAT is illustrated in Figure 1a. Briefly, a non-focused nanosecond pulsed laser illuminates the imaging region. Upon illumination, optical absorbers undergo transient thermoelastic expansion, generating ultrasonic waves (PA signals) that radiate outward. These signals are recorded by a ring-shaped transducer array surrounding the object, providing 360° full-view measurements. The acquired radio-frequency (RF) signals are then preprocessed and reconstructed using reconstruction algorithms to obtain an estimate of the initial pressure distribution.

Following laser illumination, the pressure *p*(**r**, *t*) at position **r** and time *t* in an acoustically homogeneous medium in response to a laser pulse *I*(*t*) obeys the following equation [6]:

(1) $$\nabla^{2}p(r,t)-\frac{1}{c^{2}}\frac{\partial^{2}}{\partial t^{2}}p(r,t)=-\frac{\beta }{C_{p}}\frac{\partial }{\partial t}A(r)I(t),$$

where *C_p_* is the specific heat, *A*(**r**) denotes the deposited optical energy per unit volume, and *c* is the SoS. The symbol ∇ denotes the spatial gradient operator, and ∇^2^ the corresponding Laplacian.

A full-ring transducer array is employed to acquire the PA signals. Given the recorded PA signals *p*(**r**_0_, *t*), the initial pressure at **r** can be reconstructed via BP method [7]:

(2) $${p_{0}(r)=\frac{-r_{0}^{2}}{2\pi \eta c^{4}}\oint d\theta \frac{1}{t}\frac{\partial p(r_{0},t)}{\partial t}|}_{t=\left|r_{0}-r\right|/c},$$

where *η* = *β*/*C_p_*, *β* is the isobaric volume expansion coefficient; *r*_0_ denotes the radius of the full ring array, *dθ* denotes angular increment on the ring and **r**_0_ is the spatial location of the transducer elements.

Regarding BP versus CT-style FBP. In X-ray CT, filtered back-projection (FBP) is derived from the inversion of the Radon transform, where a ramp-type filter is applied to projection data before back-projection. In PAT, however, the forward model is acoustic wave propagation rather than line integrals. Accordingly, the commonly used analytic reconstruction for homogeneous media and full-view geometries is typically written in a back-projection form, where the required “filtering” is inherently embedded as time-domain weighting and temporal differentiation operators acting on the measured pressure signals *p*(**r**_0_, *t*) [7]. Therefore, Equation (2) is already a filtered back-projection tailored to PAT.

Equation (2) assumes a constant SoS, whereas biological tissues generally exhibit spatially varying acoustic properties. Such heterogeneities introduce wavefront aberrations during acoustic propagation. Also, the mismatches between the assumed and true SoS result in pronounced reconstruction artifacts (Figure 1b). Accurate and voxel-wise determination of *c* is impractical in most biomedical settings, motivating the development of a data-driven post-processing approach to correct these SoS-induced distortions.

### 2.1. Training Dataset Preparation

Training data were prepared by 2D simulations using the k-Wave MATLAB toolbox (Version 1.3) [17]. To capture the structural diversity encountered in practical PAT, we constructed synthetic phantoms using elliptical inclusions and curved line absorbers, which approximate two prevalent morphology classes: compact targets and tubular structures [44]. Elliptical inclusions emulate focal lesions, vascular cross-sections, and other compact, blob-like targets. By sampling axis lengths and aspect ratios over a wide range, we cover both near-circular and elongated shapes typical of tumors or vessel cross-sections. Curved line sources represent tubular structures such as blood vessels, nerve bundles, and fiber tracts.

Although the training phantoms are synthetically generated, the chosen primitives are designed to capture two prevalent geometric building blocks in PAT: compact absorbers and tubular structures. From an imaging-physics perspective, SoS-induced distortions in reconstructed images are primarily driven by propagation time-of-flight errors and refraction at SoS interfaces; therefore, the resulting artifacts are strongly coupled to local boundary geometry rather than organ-specific anatomical semantics. By systematically randomizing the size, aspect ratio, orientation, curvature, amplitude, and spatial distribution of these primitives, our dataset aims to cover a wide range of morphology-dependent aberration patterns while avoiding overfitting to a single anatomy. In this study, we prioritized a controlled and highly diverse design to systematically span the major morphology classes relevant to SoS aberrations. Notably, a similar synthetic simulation strategy has been adopted in prior DL-based SoS-aberration suppression studies and shown to yield strong correction performance in linear-array PAT [35], which supports the practicality of such a design choice. Extending the training data to more anatomy-mimicking phantoms is an important direction for future work.

Randomized initial-pressure maps were created on a 600 × 600 grid (pixel size: 50 µm) as follows: (a) Elliptical sources. Major and minor axes were sampled from an exponential distribution (4–51 pixels) with aspect ratios in [0.5, 0.9] and amplitudes in [0.5, 1.0]. A scaled probability mask modulated each ellipse to introduce spatial variability, and ellipses were placed non-overlapping by enforcing minimum-distance checks. (b) Curved line sources. Curvatures were sampled at random, with lengths of 10–180 pixels, widths of 2–8 pixels, and amplitudes in [0.6, 1.0], then superimposed onto the map to increase structural complexity. (c) Zero-padding. Each 600 × 600 map was zero-padded to 1024 × 1024 pixels to accommodate numerical wave propagation.

For each synthesized pressure distribution, a full ring transducer array of radius 24 mm (360 elements) recorded PA signals at 50 MHz, outputting a 360 × 4001 RF data matrix per example. Each detector was modeled as an ideal point receiver. These paired RF datasets and corresponding ground-truth (GT) pressure maps form the basis for our two-stage curriculum learning protocol. The bandwidth of the received signal is 3–7 MHz.

To facilitate transfer learning, data generation was divided into two stages: (1) Homogeneous SoS offsets. Simulations were performed in a homogeneous medium with an acoustic absorption coefficient of 0.5 dB/(MHz·cm). For each initial-pressure map, a constant SoS was drawn sequentially from *c*(*x*) = {1460, 1470, …, 1550} m/s, covering typical tissue values in biomedical ultrasound. Repeating this for 400 maps provided an initial-pressure map set (*IPM*_1_) and the corresponding RF dataset *RF*_1_ (dimensions: 400 × 360 × 4001). Additive Gaussian noise with a maximum amplitude of −20 dB was applied to *RF*_1_. (2) Heterogeneous SoS distributions. Simulations were conducted in piecewise-constant media by partitioning each initial-pressure map into four equal-area square subregions. In each realization, the SoS for each subregion was randomly sampled from *c*(*x*), and random Gaussian noise with a maximum amplitude of −20 dB was added to the RF data.

Importantly, within the 1460–1550 m/s range, the dominant cause of degradation for BP reconstruction is the path-dependent time-of-flight (phase) mismatch and the associated refraction-driven wavefront distortion introduced by spatial SoS variations. In a full-ring geometry, the large number of source–detector pairs spanning all angles ensures that each propagation path samples different combinations and fractions of the four subregions, yielding continuously varying path-averaged effective SoS across views. Therefore, this four-region setting provides a controlled yet challenging approximation that captures the key SoS-mismatch mechanisms while enabling reproducible simulations and systematic evaluation.

**Figure 2:**
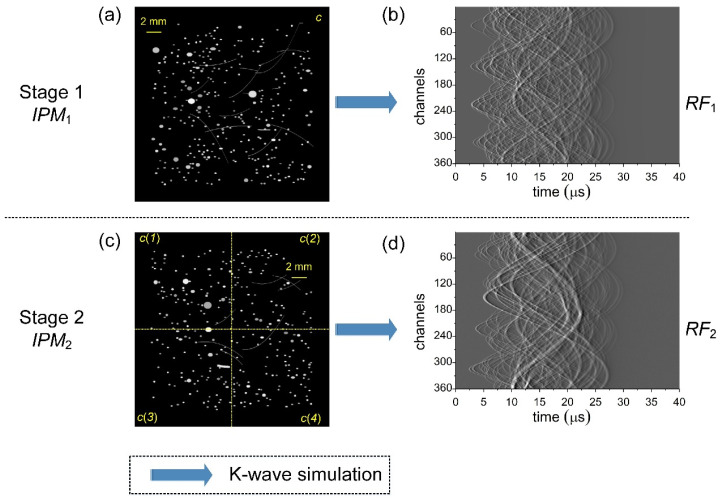
Initial-pressure maps and their corresponding RF data. (**a**) One initial-pressure map (*IPM*_1_) was used in training stage 1, where *c* denotes the homogeneous SoS. (**b**) The corresponding wavefront of (**a**). (**c**) One initial-pressure map (*IPM*_2_) used in training stage 2. The field is divided into four sub-regions by dotted yellow lines, and the SoS in each region is randomly selected from *c*(*x*), and is labeled as *c*(*1*)–*c*(*4*) in the figure. (**d**) The corresponding wavefront of (**c**).

Repeating this for 400 maps produced *IPM*_2_ and its RF dataset *RF*_2_ (400 × 360 × 4001). The initial-pressure maps and RF data used in the first and second training stages are shown in Figure 2.

### 2.2. Network Architecture and Training Strategy

We adopt a ResNet-50 backbone pretrained on ImageNet (available via Torchvision). ResNet-50 was selected over lighter encoders (e.g., ResNet-18) to better capture the complex, spatially varying distortions caused by SoS heterogeneity and measurement noise. In our preliminary tests, lightweight backbones were generally sufficient for correcting simpler uniform SoS offsets, but tended to underfit under heterogeneous SoS conditions, leading to incomplete correction. Prior to the network backbone, we implemented a pre-module that performs standard BP reconstruction in Python (Version 3.10), allowing direct conversion of RF data into PA images within an end-to-end DL workflow. The pre-module was then applied to *RF*_1_ using every SoS in *c*(*x*), producing 10 reconstructions per map: one using the true SoS and the remaining nine under mismatched conditions. This produced a reconstructed image dataset *RID*_1_ containing 4000 images. We used a constant SoS of 1500 m/s for BP reconstruction in stage 2, corresponding to the approximate average SoS of the heterogeneous medium, and an image dataset *RID*_2_ with 400 images was produced.

**Figure 3:**
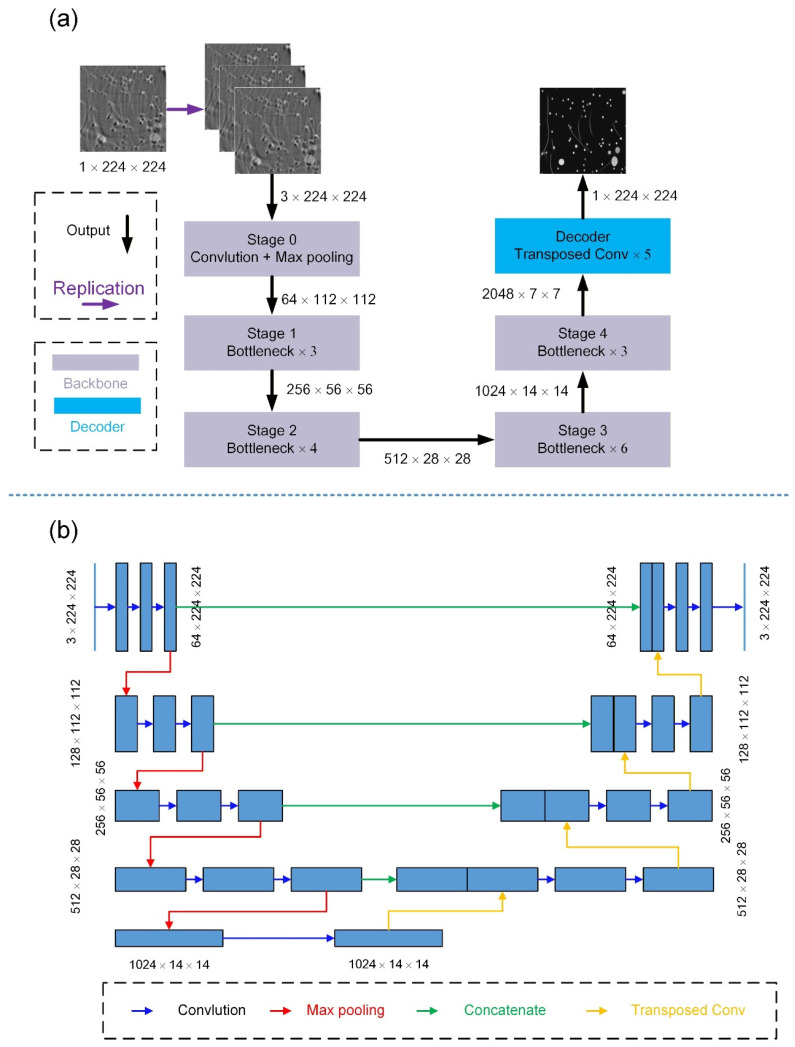
Network architectures used in this study. (**a**) TC-ResNet-50. It couples an ImageNet-pretrained ResNet-50 backbone with a five-layer deconvolutional decoder. The ResNet-50 backbone consists of an initial convolution–pooling stem (Stage 0) followed by four residual stages containing 3, 4, 6, and 3 bottleneck blocks, respectively. The decoder comprises five transposed-convolution layers that up-sample the 2048-channel feature maps to the original resolution. (**b**) U-Net baseline. It adopts a symmetric encoder–decoder structure with skip connections, each encoder block containing two convolutions with batch normalization and ReLU, followed by max pooling. The decoder mirrors this design with transposed convolutions for up-sampling and a final 1 × 1 convolution to yield a one-channel output.

Figure 3a outlines the framework of the proposed Transposed-Convolution ResNet-50 (TC-ResNet-50). To adapt the ImageNet-pretrained ResNet-50 for single-channel photoacoustic (PA) image correction, several modifications were made: (1) Each grayscale input patch was replicated to three channels to meet the input requirements of ResNet-50. (2) The original classification head was removed, retaining an initial convolution–pooling stem (Stage 0) followed by four residual stages, each containing 3, 4, 6, and 3 bottleneck blocks, respectively, as the convolutional backbone for multiscale feature extraction. (3) A decoder comprising five transposed-convolution layers was appended to up-sample the 2048-channel feature maps back to a single-channel image at the original resolution. Finally, dataset-specific normalization statistics were computed from the training data and applied consistently during both training and validation.

For benchmarking, we implemented a canonical U-Net (Figure 3b) with a symmetric encoder–decoder and skip connections. Each level uses double 3 × 3 convolutions with batch normalization and ReLU; down-sampling is by 2 × 2 max pooling and up-sampling by 2 × 2 transposed convolutions with feature concatenation. A 1 × 1 convolution produces a single-channel output. This baseline follows the canonical U-Net configuration without architectural modifications.

**Figure 4:**
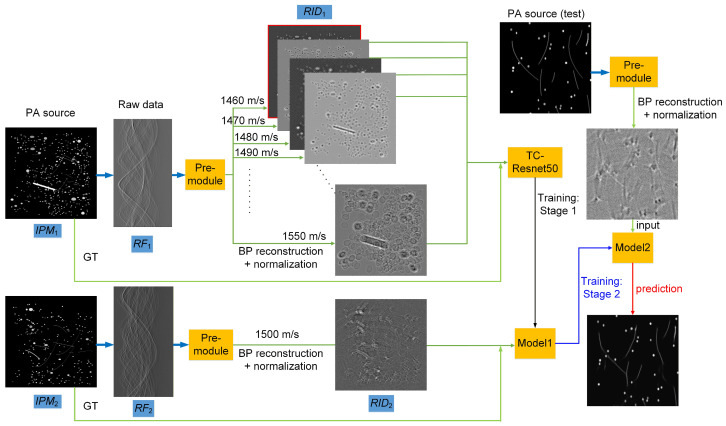
Two-Stage Training and Inference Workflow of TC-ResNet-50 for correcting SoS-induced distortions. The Pre-module performs the BP reconstruction from the recorded RF data, converting them into initial PA images that serve as the network inputs. Stage 1: pretraining on homogeneous media with uniform SoS offsets and additive Gaussian noise to learn canonical distortion and noise patterns. Stage 2: fine-tuning on heterogeneous media with random multi-region SoS variations under the same noise level.

Drawing inspiration from curriculum learning, we implemented a two-stage transfer learning protocol, as shown in Figure 4. In Stage 1, the network is pretrained on homogeneous media with a constant but intentionally mismatched SoS, and Gaussian noise (−20 dB) is added to the RF data to simulate realistic acquisition conditions. By reconstructing curves and circular absorbers under ten discrete SoS offsets, the model learns canonical artifact patterns, such as ring artifacts, radial streaks, and geometric distortions arising from uniform velocity errors. In Stage 2, the pretrained weights are fine-tuned on heterogeneous media in which SoS varies randomly across subregions, while reconstructions still assume a single global SoS. This step compels the network to recognize and correct more complex, spatially varying aberrations induced by acoustic heterogeneity.

In the first training stage, each initial-pressure map from *IPM*_1_, which was denoted as GT, was paired with its corresponding reconstructed images in *RID*_1,_ and these pairs were used to train TC-ResNet-50 (≈43 M parameters). The model’s output *P*(*RID*_1_) was compared against the GT via mean squared error (MSE):

(3) $$L=\frac{1}{N}\sum_{i=1}^{N}{\left(IPM_{1}(i)-P(RID_{1})(i)\right)}^{2},$$

where N denotes the total number of pixels across all images in the batch, *P*(•) was the prediction function of the networks. Equation (3) defines the loss function used in this work.

To satisfy the network’s 224 × 224 input requirement and augment the data, each full-resolution image was randomly cropped into four patches, and each patch was then subjected to random rotation, horizontal flipping, and amplitude scaling. Training used batches of 16 over 400 epochs and was optimized with AdamW (initial learning rate 1 × 10^−3^, weight-decay coefficient 1 × 10^−4^), and a cosine-annealing schedule (Tmax = 400, floor learning rate 1 × 10^−6^). The best model at convergence, determined by an early-stopping criterion (minimum validation loss), was denoted *M*_1_. For the second stage, *IPM*_2_ and *RID*_2_ were paired in the same manner to fine-tune *M*_1_ under the same training regime, except for extending the cosine schedule to Tmax = 2000. The final converged model is referred to as *M*_2_.

For fair comparison, the benchmark U-Net (≈31 M parameters) was trained using the same two-stage curriculum strategy as TC-ResNet-50. In the first stage, the network was pretrained on homogeneous SoS offsets to learn canonical artifact patterns, and in the second stage, it was fine-tuned on heterogeneous SoS distributions. All training parameters, including optimizer (AdamW), learning rate schedule (cosine annealing), batch size, early-stopping criterion and data augmentations, were kept identical to those used for TC-ResNet-50.

## 3. Results

### 3.1. In Silico Experiments

We first evaluated TC-ResNet-50’s performance under controlled simulation conditions. A dataset of 400 virtual phantoms (280 training, 80 validation, and 40 test) was generated as described in Section 2.1. All models were trained and tested on an Ubuntu workstation (Intel Core i7 CPU, 24 GB RAM, NVIDIA RTX 4070 Ti GPU).

**Figure 5:**
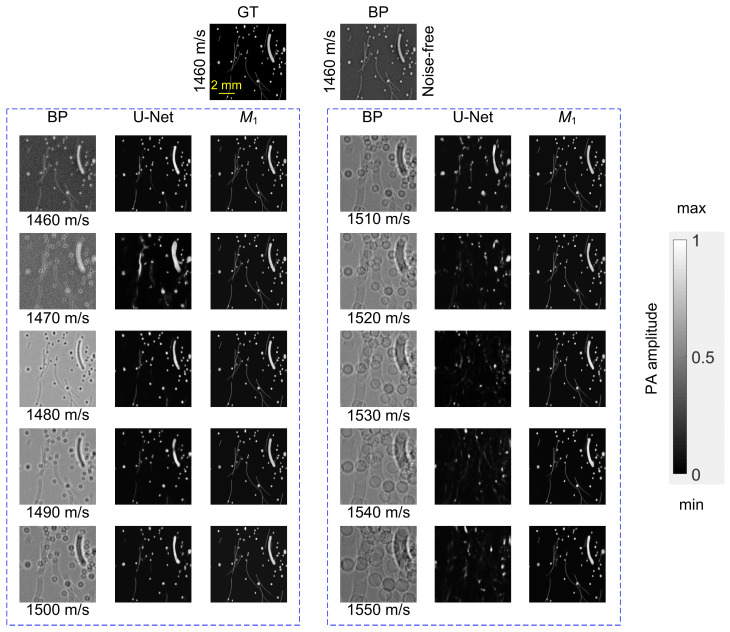
Performance of TC-ResNet-50 (*M*_1_) in correcting SoS mismatch artifacts under homogeneous conditions. The two top images represent the GT pressure distribution (**left**) and the BP reconstruction using the true SoS (**right**). The dashed boxes show a comparison of the outputs from BP, U-Net, and *M*_1_ (from (**left**) to (**right**)), with each row corresponding to a different reconstruction SoS ranging from 1460 m/s to 1550 m/s.

The RF dataset *RF*_1_ was reconstructed by the pre-module with BP method using SoS offsets from 0 to ±90 m/s. As illustrated in Figure 5, BP reconstructions suffer from severe ring-shaped artifacts, blurred inclusions, and geometric distortions. Moreover, the BP reconstruction worsens with increasing mismatch.

The BP reconstructed images and their GT images were then fed into TC-ResNet-50. In Stage 1, the network learned to correct canonical artifacts arising from BP under uniform SoS mismatches. After 400 epochs, the converged Model *M*_1_ demonstrated substantial artifact suppression in homogeneous media, as shown in Figure 5. Even with SoS offsets up to 90 m/s, *M*_1_ effectively restored image fidelity.

**Figure 6:**
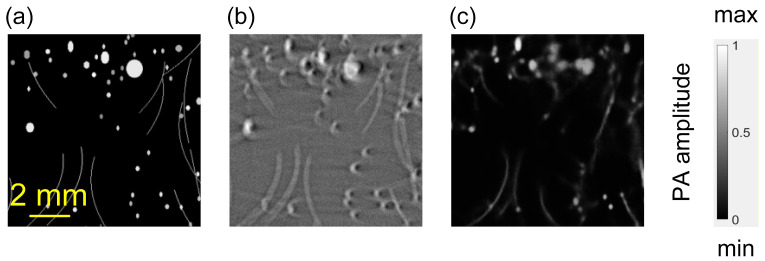
Output of *M*_1_ on heterogeneous numerical model. (**a**) GT of the heterogeneous numerical model. (**b**) BP reconstruction. (**c**) Output of *M*_1_.

However, when the *M*_1_ model trained on homogeneous SoS offsets was directly applied to heterogeneous SoS scenarios, it failed to generalize and produced severe distortions, as illustrated in Figure 6. Moreover, the training did not converge reliably regardless of whether the network was trained solely on heterogeneous SoS data or jointly on both homogeneous and heterogeneous SoS data. These observations indicate that the heterogeneous SoS correction task is too complex for single-step training to learn directly from highly variable data distributions. Therefore, the proposed two-stage curriculum is essential for stable convergence and generalization.

For benchmarking, we trained the U-Net on the same datasets and hardware under the training protocol mentioned in Section 2.2. The U-Net architecture employed here follows the configuration illustrated in Figure 3b. The training of the U-Net on *RID*_1_ converged after approximately 900 epochs.

The BP, U-Net and TC-ResNet-50 models were then evaluated across the full range of SoS mismatch scenarios. Table 1 summarizes the mean ± SD of mean squared error (MSE), structural similarity index measure (SSIM), and Pearson correlation coefficient (PCC) over the simulated test images.

MSE quantifies the average pixel-wise intensity deviation from the GT, with values ranging from 0 (perfect agreement) to 1 (maximal error across a fully normalized [0, 1] image).

SSIM is defined as:

(4) $$\mathrm{SSIM}(x,y)=\frac{\left(2\mu_{x}\mu_{y}+C_{1})(2\sigma_{xy}+C_{2}\right)}{\left(\mu_{x}^{2}+\mu_{y}^{2}+C_{1})(\sigma_{x}^{2}+\sigma_{y}^{2}+C_{2}\right)},$$

where *x* and *y* denote the predicted image *P*(*RID*_1_) and the GT image *IPM*_1,_ respectively. *µ_x_* and *µ_y_* represent their mean pixel intensities, *σ_x_* and *σ_y_* their standard deviations, and *σ_xy_* the covariance between *x* and *y*. *C*_1_ = 0.01 and *C*_2_ = 0.03 are small stabilizing constants. SSIM assesses perceptual and structural fidelity, with a theoretical range of −1 to 1 (where 1 denotes identical structure, 0 no correlation, and negative values an inverse relationship), but in most imaging contexts falls between 0 and 1.

The PCC is defined as:

(5) $$\mathrm{PCC}=\frac{\sum_{i=1}^{N}\left(P(RID_{1})(i)-\bar{P})(IPM_{1}(i)-\bar{G}\right)}{\sqrt{\sum_{i=1}^{N}{\left(P(RID_{1})(i)-\bar{P}\right)}^{2}\sum_{i=1}^{N}{\left(IPM_{1}(i)-\bar{G}\right)}^{2}}},$$

where $\bar{P}$ and $\bar{G}$ are mean pixel intensities of *P*(*RID*_1_) and *IPM*_1_, respectively. The PCC measures the linear relationship between reconstructions and GTs, also ranging from –1 to 1; values closer to 1 indicate stronger agreement in overall intensity patterns and contrast.

The low MSE, high SSIM and PCC achieved by *M*_1_ demonstrate its better restoration performance over both BP and U-Net.

In training stage 2, we synthesized 400 simulated phantoms with four randomly assigned SoS regions and added Gaussian noise up to −20 dB during RF data excitation, as shown in Figure 2c,d. After being processed by the pre-module, the noisy BP-reconstructed images (*RID*_2_) were paired with the corresponding GTs (*IPM*_2_) and used to further train *M*_1_, resulting in the fine-tuned model *M*_2_ after approximately 1000 epochs (model size ≈ 164 MB). This stage was designed to teach the network to correct the spatially varying distortions induced by heterogeneous SoS distributions. Similarly, the U-Net underwent fine-tuning initialized from its Stage 1 weights, yielding the best performance at around 2300 training epochs.

**Figure 7:**
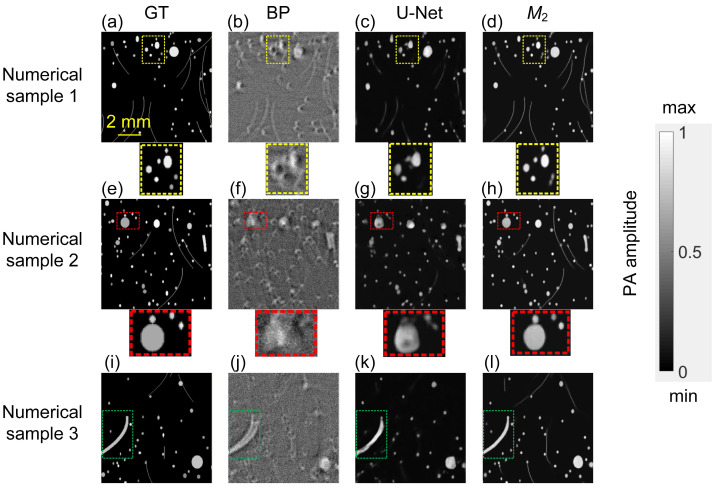
Reconstruction comparison on numerical test samples. (**a**–**d**) Sample 1: (**a**) GT; (**b**) BP reconstruction; (**c**) Output of U-Net; (**d**) Output of *M*_2_. Insets highlight some small circular inclusions (yellow). (**e**–**h**) Sample 2: (**e**) GT; (**f**) BP reconstruction; (**g**) Output of U-Net; (**h**) Output of *M*_2_. Insets (red) focus on the largest spherical absorber. (**i**–**l**) Sample 3: (**i**) GT; (**j**) BP reconstruction; (**k**) Output of U-Net; (**l**) Output of *M*_2_. Insets (green) emphasize the curved linear absorber.

Figure 7 compares the proposed method with BP algorithm (reconstructed at SoS = 1500 m/s) and U-Net under the combination of SoS heterogeneity and noise. As shown, BP reconstructions exhibit severe artifacts and blurred structures. In heterogeneous SoS scenarios, distortions themselves become direction-dependent. Circular or elliptical sources may appear as hollow rings (yellow dashed box), or irregular blobs (red dashed box). A U-Net trained under the same heterogeneous conditions delivers modest improvement over BP: while it attenuates some distortions, it frequently loses some information. By contrast, *M*_2_ restores meaningful features across all test cases and consistently recovers the original object geometry. Moreover, spatially varying aberrations within a single feature, such as a curved line that is only mildly warped at one end but severely distorted at the other (green dashed box), pose a particular challenge. Our two-stage curriculum enables the network to first learn artifact patterns associated with uniform SoS mismatch and then adapt to more complex, spatially heterogeneous distortions, thereby improving spatially adaptive correction. As a result, *M*_2_ reconstructs the entire curved structure with a smooth intensity profile that closely matches the GT, indicating improved robustness and reconstruction fidelity.

**Figure 8:**
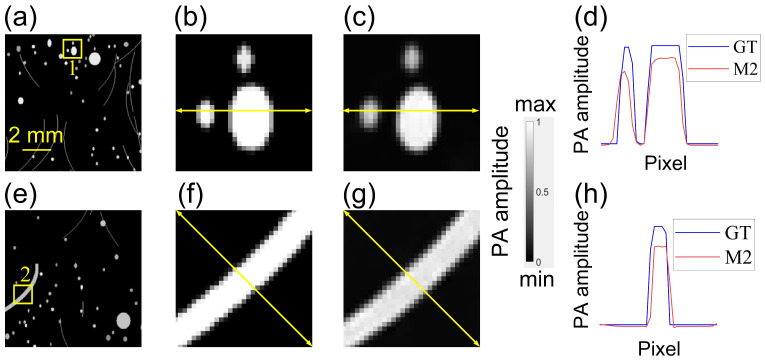
Cross-sectional intensity profiles comparing GT and *M*_2_ reconstructions in two local regions. (**a**) Full reconstructed image with Region 1 (yellow box) highlighted. (**b**) GT sub-image extracted from Region 1. (**c**) *M*_2_ output sub-image for Region 1 (identical intensity window as in (**b**)). (**d**) Normalized horizontal intensity profile (yellow arrow) through the two circular absorbers in (**b**,**c**). (**e**) Full reconstructed image with Region 2 (yellow box) highlighted. (**f**) GT sub-image extracted from Region 2 (curved absorber). (**g**) *M*_2_ output sub-image for Region 2. (**h**) Normalized diagonal intensity profile (yellow arrow) through the curved absorber in (**f**,**g**).

Figure 8 gives the intensity profiles along the cross-section of local regions to further examine the fine details recovered by *M*_2_. In Region 1 (Figure 8a–c), the circular absorbers reconstructed by *M*_2_ closely match the GT, with only minimal blurring. The normalized horizontal profiles (Figure 8d) exhibit nearly identical full-width at half-maximum (FWHM), differing by at most 1–2 pixels, indicating preservation of both contrast and spatial resolution. In Region 2 (Figure 8e–g), a curved absorber distorted by heterogeneous SoS is almost closely recovered by *M*_2_. Its diagonal intensity profile (Figure 8h) shows a peak-position deviation of less than two pixels, demonstrating quantitative accuracy alongside visual fidelity.

To enable a more accurate comparison in the quantitative analysis, the optimal reconstruction SoS for each of the 40 *RF*_2_ test datasets was identified via minimum MSE selection. BP images were then reconstructed at the optimal SoS, followed by generation of the corresponding outputs from *M*_2_ and the U-Net. Quantitative evaluation across the entire test set, using MSE, SSIM, and PCC, verifies these observations (Table 2).

Table 2 summarizes the mean ± standard deviation of MSE, SSIM, and PCC over the 40 test images under heterogeneous SoS conditions. Both learning-based methods outperform the BP reconstruction. *M*_2_ further achieves lower MSE, higher SSIM, and stronger PCC compared with both BP and U-Net, demonstrating enhanced robustness. Although an optimal single SoS was selected for each dataset via minimum-MSE, the underlying SoS distribution is spatially heterogeneous. When the SoS contrast between regions is large, a single global SoS can only provide a compromise, leaving residual spatially varying time-of-flight errors and refraction effects that affect different structures to different extents. This contributes to the larger inter-sample variability observed for BP.

To make the error level more explicit, we summarize the performance of the initial reconstructions (BP) and the corrected outputs (TC-ResNet-50) using MSE/SSIM/PCC. In Table 1, *M*_1_ reduces MSE by ~98.2% (0.28 → (5 ± 2) × 10^−3^), while increasing SSIM by +0.69 (0.22 → 0.91, ~+314% relative) and increasing PCC by +0.66 (0.26 → 0.92, ~+254% relative). In Table 2, *M*_2_ reduces MSE by ~91.4% (0.14 → (1.2 ± 0.4) × 10^−2^), with SSIM improved by +0.87 (0.03 → 0.90) and PCC improved by +0.79 (0.10 → 0.89).

Beyond the overall performance ranking, the numerical differences in Table 1 and Table 2 also carry clear practical implications for SoS aberration correction in full-ring PAT. The reduction in MSE achieved by TC-ResNet-50 indicates more accurate recovery of absorber energy concentration, suggesting effective suppression of SoS-induced energy spreading and geometric distortion. Meanwhile, the increase in SSIM reflects improved preservation of local structural patterns, which is particularly important for resolving thin or elongated absorbers that are highly sensitive to SoS mismatch. The consistently high PCC further demonstrates that the proposed method better maintains the global spatial correlation with the GT, indicating reduced spatial warping across the imaging field. In contrast, although U-Net improves average image quality compared with BP, its relatively lower SSIM/PCC and larger variance suggest limited robustness under strong SoS heterogeneity. Collectively, these quantitative improvements confirm that TC-ResNet-50 provides not only higher numerical accuracy but also more stable and physically meaningful correction of SoS-induced artifacts in full-ring PAT.

### 3.2. Phantom Experiments

**Figure 9:**
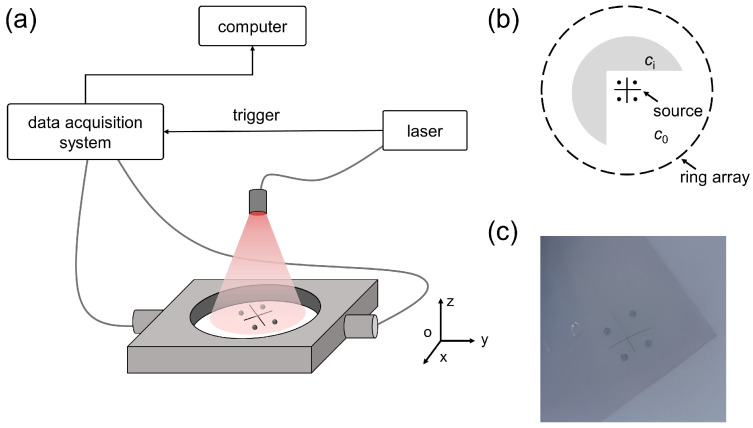
Phantom experiment setup. (**a**) Schematic diagram of the PAT system with a ring-shaped transducer array. (**b**) Schematic diagram of the experimental sample structure. (**c**) Photograph of the sample.

To validate the performance of our method in practical settings, we conducted phantom experiments. The experimental setup is illustrated in Figure 9a. A Q-switched Nd:YAG laser (OPOTEK LLC, Carlsbad, CA, USA) operating at 780 nm served as the excitation source, delivering 4.5 ns pulses at a repetition rate of 10 Hz. A custom-designed ring-shaped array transducer (Doppler Electronic Technologies, Guangzhou, China) with a central frequency of 5 MHz and 70% bandwidth was employed for signal acquisition. The ring-shaped transducer had a diameter of 4.8 cm and 256 channels. An ultrasonic acquisition system (Custom-designed, Nanjing, China) was used to record the signals at a sampling frequency of 50 MHz.

In principle, shorter laser pulses provide broader excitation spectra and better image resolution; however, in our MHz-band full-ring system the effective received bandwidth is primarily limited by the transducer frequency response and acoustic attenuation, so nanosecond pulse-width variations have only a minor impact on the effective bandwidth/image resolution and are not expected to qualitatively change the SoS-induced aberrations.

Phantom experiments used the same optical absorber configuration-two 0.15 mm tungsten wires (Ningbo Zhuogu New Materials, Ningbo, China) and four 1 mm iron spheres (Zhuoyue Alloy, Guangdong, China) fixed at the ring center—while varying only the surrounding medium to create two distinct samples. Both phantoms comprised a low-SoS region (2% agarose (Foshan Krypai Chemical, Foshan, China), SoS ≈ 1520 m/s) and a high-SoS region. In Sample 1, the high-SoS region was prepared by mixing 2% agarose solution with glycerol (Foshan Krypai Chemical, Foshan, China) at a 9:1 ratio (SoS ≈ 1560 m/s), whereas in Sample 2, the ratio was 7:1 (SoS ≈ 1600 m/s). To fabricate phantoms with different SoS in the high-SoS region, we first prepared two high-SoS media by mixing 2% agarose with glycerol at the two ratios above. Each mixture was poured into a high-precision 3D-printed Teflon mold and allowed to cool until solidified. The resulting high-SoS block was then placed in a standard glass beaker (diameter: 4 cm; height: 2 cm), after which 2% agarose (low SoS) was poured to cast the surrounding region. When the liquid level reached half of the mold scale, the absorber assembly (tungsten wires and iron spheres), pre-fixed in solidified 2% agarose, was placed into the mold and gently pressed to keep it at the desired height. The casting with 2% agarose was then continued until the mold reached its maximum scale. Finally, the phantom was allowed to cool at room temperature. After data acquisition for the first phantom, the sample was carefully removed, and the entire casting procedure was repeated using the other high-SoS medium to produce the second phantom. In this way, two phantoms were obtained for controlled experiments.

The elevation height of the sample was set relative to the imaging plane of the ring array, and the position of the absorbers within the plane was confined to a region 15 mm in the center of the annular array.

**Figure 10:**
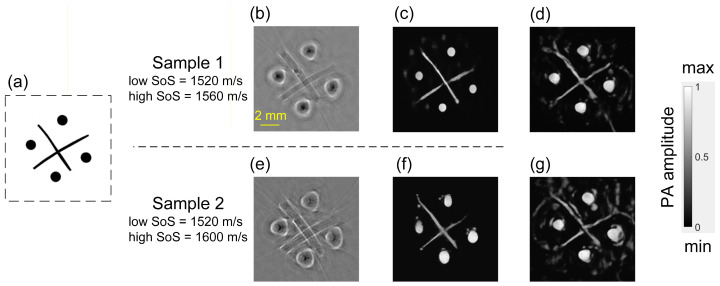
Experimental phantom results and *M*_2_ outputs. (**a**) Enhanced binary picture of Figure 9c. (**b**) BP reconstruction of sample 1. (**c**) Output of *M*_2_ for (**b**). (**d**) Output of U-Net for (**b**). (**e**) BP reconstruction of sample 2. (**f**) Output of *M*_2_ for (**e**). (**g**) Output of U-Net for (**e**).

Figure 9b,c shows the medium layout and a photograph of the assembled phantom. In Figure 9b, the shaded area labeled “*c*_i_” denotes the high SoS region, while the area labeled “*c*_0_” represents the low SoS region. During imaging, a z-axis translation stage aligned the absorbers to the transducer’s focal plane. To obtain the binary GT of the phantom, we converted Figure 9c to a grayscale image, where the difference in intensity values was used to distinguish the optical absorbers (high values) from the surrounding medium (low values). The grayscale image was then binarized to obtain a black-and-white image (Figure 10a). After laser excitation, PA data were acquired; BP reconstructions used visually selected SoS values (1520 m/s for sample 1, 1525 m/s for sample 2). The BP outputs are shown in Figure 10b,e, and the subsequent *M*_2_ results in Figure 10c,f. In Figure 10b,e, BP exhibits strong artifacts around spheres and fragmented, blurred wire segments due to heterogeneous SoS. In Figure 10c,f, *M*_2_ produces uniformly bright circular inclusions and continuous, sharply defined wires. U-Net outputs (Figure 10d,g) improve on BP but retain noticeable artifacts and distortions.

Simultaneously, the quality of the images in Figure 10c,d,f,g is quantified by the MSE, SSIM, and PCC, with Figure 10a serving as the GT. As summarized in Table 3, it can be observed that in the experimental scenario, where the maximum SoS difference in the medium is 80 m/s, *M*_2_ demonstrates strong performance; it consistently outperforms U-Net across both samples, indicating more reliable contour recovery.

### 3.3. In Vivo Experiments

**Figure 11:**
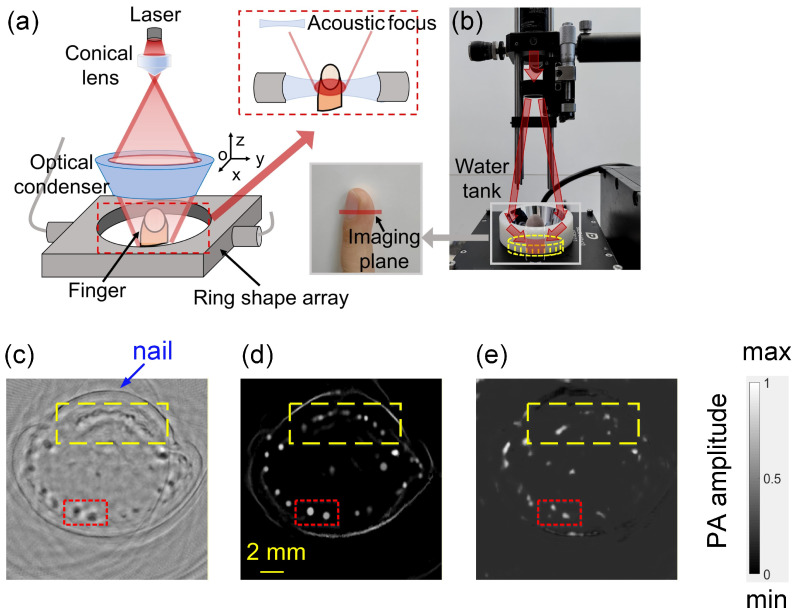
Experimental setup of the PAT system. (**a**) Schematic of the PAT system. The red dashed box depicts a cross-sectional view of the confocal design. (**b**) Photograph of the experimental setup. The gray solid box indicates the region of the measured finger. (**c**) BP reconstruction. The blue arrow indicates the fingernail, while the yellow and red dashed boxes highlight the subungual capillary network beneath the nail bed and the two main blood vessels in the fingertip region, respectively. (**d**) Output of *M*_2_. (**e**) Output of U-Net.

We conducted an in vivo experiment on human finger to verify the effectiveness of the method on biological tissues. As shown in Figure 11a, a conical lens (JCOPTIX, Nanjing, China) and an optical condenser (JCOPTIX, Nanjing, China)were utilized to focus the laser onto the acoustic focus area. The transducer array was the same as that used in the phantom experiments. The array was immersed in a water tank (Custom-designed, Nanjing, China), and the finger was positioned at the center of the array. Considering the SoS variations in the fingernail and bone, we performed imaging on the fingertip. The same laser system as in the phantom experiments was used here, operating at 780 nm with a pulse duration of 4.5 ns and a repetition rate of 10 Hz. The measured maximum laser exposure on the tissues was 15 mJ/cm^2^, which was within the ANSI safety limit. Figure 11b presents a photograph of the experimental system.

The imaging region of the finger is illustrated in the inset of Figure 11b. Due to the presence of the nail, this region can be regarded as an acoustically heterogeneous medium. After the system acquired the RF data, PAT images were reconstructed using the BP algorithm, *M*_2_ and the U-Net, respectively. Upon visual inspection, 1535 m/s was selected as the optimal SoS for BP reconstruction, and the result is shown in Figure 11c. The *M*_2_-optimized reconstruction is displayed in Figure 11d, and the U-Net reconstruction in Figure 11e. As observed, *M*_2_ effectively suppresses the background noise present in the BP reconstruction, and the previously blurred vascular cross-sections become sharply defined, particularly the complex subungual vascular network indicated by the yellow dashed box and the two main vessels in the fingertip region indicated by the red dashed box. The image reconstructed by *M*_2_ shows structural similarity to previously reported finger PAT results [45]. In contrast, although the U-Net also suppresses background noise in the BP reconstruction it tends to lose more fine biological structural information.

### 3.4. Computational Performance

Inference was carried out on the same desktop workstation described in Section 3.1, namely, an Intel Core i7 CPU with 24 GB RAM and an NVIDIA GeForce RTX 4070 Ti GPU. For inference on a single 224 × 224 image, U-Net achieves 18 ms with a peak GPU memory usage of 326 MB, whereas TC-ResNet-50 takes 17 ms with a peak GPU memory usage of 347 MB (BP pre-module excluded). This result demonstrates that our TC-ResNet-50 framework meets real-time requirements. Such throughput is sufficient for in vivo full-ring PAT imaging, enabling artifact-corrected reconstructions at video-rate speeds. This high frame rate underscores the practical feasibility of deploying the proposed method in real-world applications.

## 4. Conclusions

This study shows that a modified ImageNet-pretrained ResNet-50 architecture (TC-ResNet-50) can effectively correct reconstruction artifacts caused by inhomogeneous SoS distributions in full-ring PAT. By employing a two-stage curriculum, initially training on homogeneous media BP artifacts under varied SoS mismatches, then fine-tuning on simulated heterogeneous SoS fields, our model effectively learns both uniform-error patterns and complex spatially varying distortions. The two-stage curriculum was found necessary: *M*_1_ is inapplicable to heterogeneous SoS data, and single-stage training directly on heterogeneous data did not converge reliably. In numerical experiments, TC-ResNet-50 yields a substantial reduction in MSE and consistent improvements in SSIM and PCC relative to BP. In phantom experiments with SoS difference up to 80 m/s, *M*_2_ essentially restores absorber shapes from BP reconstructions. During in vivo finger imaging, *M*_2_ likewise suppresses SoS-induced artifacts and better delineates vascular structures than BP and U-Net, indicating robust generalization to tissue heterogeneity. Note that, although we trained solely on numerical simulation data, the network still performed well in both phantom and in vivo experiments, demonstrating its generalizability.

The effectiveness of TC-ResNet-50 in this scenario might be attributed to three factors. First, its ImageNet-trained backbone provides rich, transferable low-level filters that facilitate recognition and correction of complex PA artifacts from limited domain-specific samples. Second, the residual architecture facilitates stable gradient flow when learning the subtle mapping from BP artifacts to true pressure distributions; skip connections allow the network to directly transfer clean, high-frequency information from input to output, while intermediate layers focus on modelling the artifact corrections themselves. Third, the two-stage curriculum learning protocol exposes the network to increasingly complex distortions and enhances its ability to generalize to spatially varying aberrations.

However, there remains room for further improvement in our work. When a SoS mismatch is present during reconstruction, a larger mismatch causes the energy of optical absorbers to spread over a wider area in the image, leading to lower energy per unit area. When the noise level exceeds −20 dB, in cases of severe mismatch, the absorber energy can be overwhelmed by noise, making the absorbers difficult to identify and consequently degrading model performance—especially for thin, line-shaped absorbers. In addition, the current study is limited to 2D circular full-ring configurations; the generalization of the proposed model to non-circular ring arrays and 3D imaging setups remains to be investigated in future work. Finally, we have not yet conducted dedicated experiments with continuous SoS gradients. Physically, both piecewise-constant and continuous-gradient SoS mismatches primarily introduce spatially varying time-of-flight errors, leading to distortion and energy spread. In future work, this framework could be extended by augmenting the training set with continuously varying SoS maps and fine-tuning the network accordingly.

In summary, this work demonstrates that a transfer learning-based framework, combining a ResNet-50 backbone with a tailored decoder and a two-stage curriculum, can markedly mitigate the SoS-induced aberrations in full-ring PAT. The experiments show effective correction of both uniform and spatially varying distortions, thereby improving reconstruction quality under heterogeneous SoS conditions and potentially facilitating broader use of full-ring PAT in practical settings.

## Figures and Tables

**Table 1:** Test-Set Performance Comparing TC-ResNet-50 (*M*_1_) with BP and U-Net.

	BP	U-Net	TC-ResNet-50 (*M*_1_)
MSE	0.28 ± 0.16	0.12 ± 0.04	(5 ± 2) × 10^−3^
SSIM	0.22 ± 0.14	0.50 ± 0.22	0.91 ± 0.07
PCC	0.26 ± 0.39	0.59 ± 0.23	0.92 ± 0.03

**Table 2:** Test-Set Performance Comparing TC-ResNet-50 (*M*_2_) with BP and U-Net.

	BP	U-Net	TC-ResNet-50 (*M*_2_)
MSE	0.14 ± 0.07	0.02 ± 0.01	(1.2 ± 0.4) × 10^−2^
SSIM	0.03 ± 0.01	0.50 ± 0.18	0.90 ± 0.04
PCC	0.10 ± 0.10	0.44 ± 0.21	0.89 ± 0.04

**Table 3:** Quantitative comparison of phantom experiments.

	Sample 1	Sample 2
MSE	0.26 (*M*_2_) 0.39 (U-Net)	0.31 (*M*_2_) 0.43 (U-Net)
SSIM	0.77 (*M*_2_) 0.49 (U-Net)	0.70(*M*_2_) 0.47 (U-Net)
PCC	0.81 (*M*_2_) 0.46 (U-Net)	0.77 (*M*_2_) 0.41 (U-Net)

## Data Availability

The raw data supporting the conclusions of this article will be made available by the authors on request.
